# Biomimetic Superwetting Polysaccharide-Based Composite Hydrogel Interfaces from an Eco-Dialectical Perspective: Polymer Network Design, Hydration-Layer Stabilization, and Structure–Performance Relationships

**DOI:** 10.3390/polym18161952

**Published:** 2026-08-09

**Authors:** Lisha Hou, Shiyu Huang

**Affiliations:** 1 College of State Governance, Southwest University, Chongqing 400715, China; 2 College of Materials, Chemistry and Chemical Engineering, Chengdu University of Technology, Chengdu 610059, China

**Keywords:** biomimetic superwetting interface, hydration layer, antifouling, biofilm prevention, biomaterial-associated infection, structure–performance relationship, dialectics of nature

## Abstract

Biomaterial-associated infection remains a persistent challenge for implantable devices, catheters, wound dressings, and tissue-engineering scaffolds. This structured narrative review critically evaluates biomimetic superwetting polysaccharide-based composite hydrogel interfaces based on chitosan, alginate, hyaluronic acid, cellulose/nanocellulose, bacterial cellulose, and dextran. The analysis links polymer network design and cross-linking strategy to pore architecture, swelling, mechanical properties, hydration-layer stabilization, protein adsorption, bacterial adhesion, biofilm development, and cytocompatibility. Stable interfacial hydration can increase the energetic penalty for protein and bacterial approach, but high water uptake alone is insufficient: excessive swelling, low network density, poorly controlled pore interconnectivity, and weak wet-state fixation can compromise durability or provide protected sites for bacterial retention. Study-level comparisons therefore emphasize reported values for network structure, swelling, mechanics, wettability or hydration, and antibacterial/antibiofilm performance, with unreported parameters identified as such. Notably, interactions among biomaterials, bacteria, and host tissues exhibit synergistic and co-evolutionary characteristics, forming a dynamically evolving microecological balance. This eco-synergistic perspective provides a useful conceptual framework for proposing antifouling strategies that aim to regulate rather than eradicate bacterial colonization. Future work should prioritize eco-synergistic design, durable hydration, mechanically stable and porous-yet-cleanable networks, selective interfacial regulation, dynamic characterization, standardized testing, and manufacturable formulations with the minimum necessary active components.

## 1. Introduction

Biomaterial-associated infections have long limited the safe use of medical materials such as implants, catheters, wound dressings, tissue-engineering scaffolds, and interventional devices. Unlike infections dominated by planktonic bacteria, biomaterial-associated infection is not a single bacterial-proliferation event but a dynamic interfacial process governed by continuous interactions among the material surface, adsorbed proteins, microorganisms, the host response, and the local microenvironment [1,2,3,4]. After implantation or application, the material surface is rapidly exposed to complex biological environments containing blood or tissue fluid, proteins, cells, microorganisms, and metabolites. Nonspecific adsorption of proteins and other biomolecules often initiates interfacial contamination and the infection cascade. Bacteria then establish initial contact with the material surface through fimbriae, flagella, surface-adhesion proteins, hydrophobic interactions, and electrostatic interactions, and gradually form microcolonies and mature biofilms [5,6].

Traditional anti-infective strategies rely mainly on systemic antibiotics, local antimicrobial release, or contact-killing surfaces. Although these approaches can reduce bacterial burden in the short term, they are limited by poor drug penetration, passivation of active sites, retention of dead bacteria, selective pressure for drug resistance, and insufficient long-term stability in biomaterial-associated biofilm infections [7,8,9,10,11]. For medical materials in prolonged contact with tissues or body fluids, a short-term bactericidal effect is not equivalent to a sustained anti-infective effect. If protein fouling and bacterial re-adhesion continue on the material surface, the initial bactericidal advantage may not translate into long-term clinical effectiveness.

**Figure 1:**
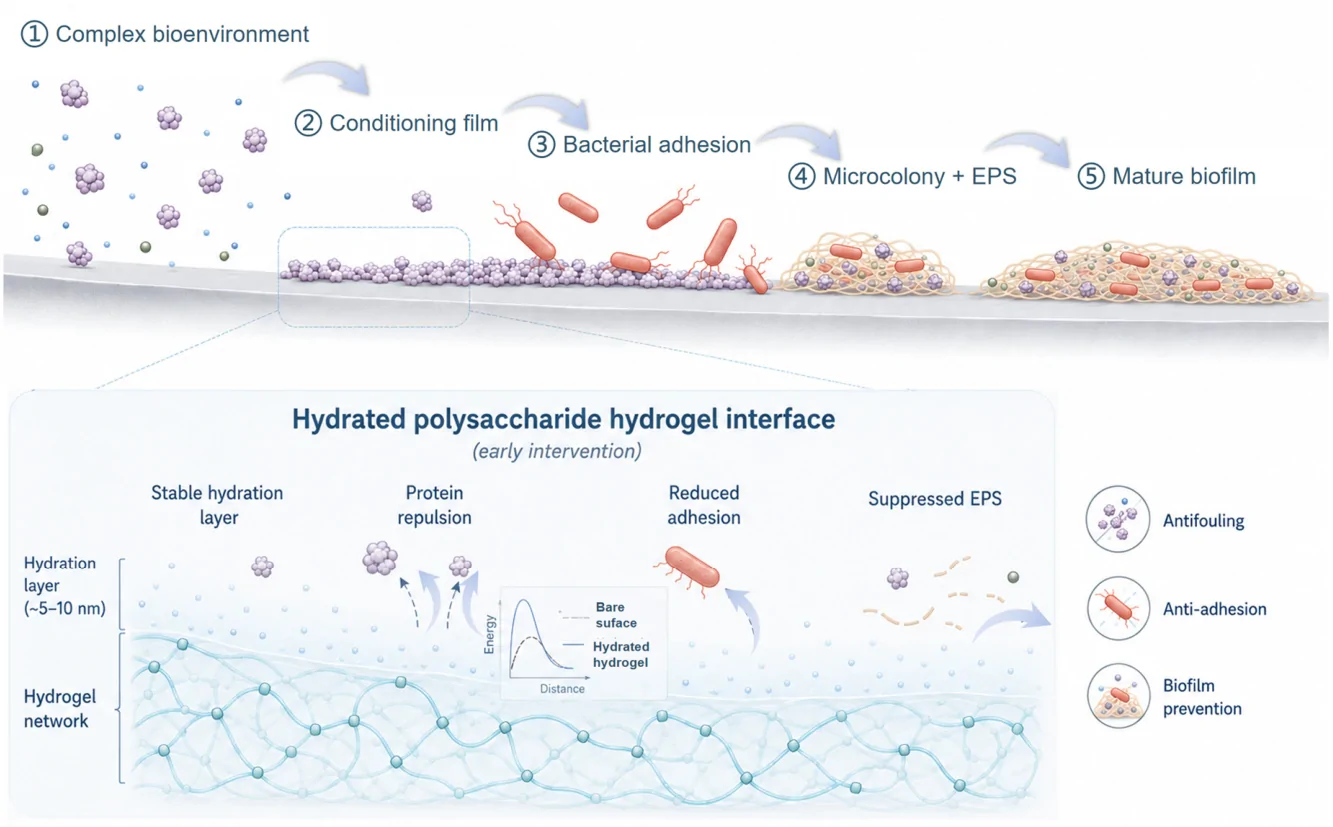
The interfacial evolution process of biomaterial-associated infections and the early intervention window of biomimetic superwetting polysaccharide-based composite hydrogels. The numbered circles and blue arrows indicate the sequential progression from the complex bioenvironment to mature biofilm formation. Purple particles represent adsorbed proteins, orange rods represent bacteria, and beige aggregates represent the EPS-rich biofilm matrix. The blue dashed line delineates the hydrated interfacial region, while the circled symbols on the right indicate antifouling, anti-adhesion, and biofilm-prevention effects. EPS, extracellular polymeric substances.

Therefore, anti-infective material design should shift from reliance on a single antibacterial mechanism to a comprehensive strategy that emphasizes early-stage blocking, interfacial regulation, and long-term stability [12]. Surface antifouling and anti-adhesion constitute an early step in infection prevention and control. If a material reduces the fouling burden during protein adsorption and initial bacterial adhesion, the infection cascade can be disrupted before biofilm maturation. Compared with strongly bactericidal strategies, early antifouling and resistance to bacterial adhesion emphasize minimal biological disturbance, limited residue, and sustained interfacial cleanliness [13]. The interfacial evolution of biomaterial-associated infection and the early intervention window provided by hydrated polysaccharide hydrogel interfaces are illustrated in Figure 1. 

Polysaccharide-based composite hydrogels provide an important materials platform for this design shift. Polysaccharides commonly contain hydrophilic functional groups such as hydroxyl, carboxyl, amino, acetamido, or sulfate groups, which bind water through hydrogen bonding, electrostatic hydration, or dipole–dipole interactions. These high-water-content polymer networks not only provide favorable wet-state conformability and tissue compatibility but can also establish a hydration layer on the material surface, thereby increasing the energetic penalty for proteins and bacteria approaching the interface [14]. However, conventional hydrophilic hydrogels are not inherently equivalent to biomimetic superwetting antifouling interfaces. Polysaccharide hydrogels function as biomimetic superwetting anti-infective interfaces only when surface chemistry, micro-/nanostructure, and polymer network hydration jointly maintain a stable water layer.

Drawing on the dialectical emphasis on the interconnected and dynamic character of natural systems described by Engels [15], this review adopts a limited dialectical-materialist view of nature to holistically examine the dynamic interactions among polymer networks, hydration layers, microorganisms, host tissues, and local biological environments. All mechanistic interpretations and material design recommendations are grounded in experimentally testable polymer and interface science.

The term “biomimetic” in this review does not imply simple replication of natural morphology; rather, it refers to extracting core mechanisms of interfacial hydration, antifouling, lubrication, wet-state adhesion, and tissue adaptation and translating them into engineerable, testable, and translatable polysaccharide-based polymer networks. Rather than treating polysaccharide hydrogels primarily as wound dressings, drug-delivery systems, or antibacterial composites, this review considers them as biomimetic superwetting anti-infective polymer interfaces and focuses on relationships among molecular structure, cross-linked networks, swelling behavior, interfacial wettability, hydration-layer stability, antifouling, and resistance to bacterial adhesion.

Accordingly, the review uses polymer network structure, hydration-layer stability, porous architecture, interfacial characterization, and application-specific evidence as its scientific basis.

## 2. Literature Search and Review Methodology

### 2.1. Review Scope and Literature Selection Strategy

This article is a structured narrative review supported by targeted searches of Web of Science, Scopus, and PubMed. Search terms combined polysaccharide hydrogel types (chitosan, alginate, hyaluronic acid, cellulose or nanocellulose, bacterial cellulose, and dextran) with interface phenomena (superwetting, hydration layer, antifouling, protein adsorption, bacterial adhesion, biofilm, and biomaterial-associated infection) and application formats (coating, wound dressing, implant, catheter, and tissue-engineering scaffold). No formal publication-year restriction was applied; recent original studies were prioritized for material performance and application evidence, while earlier studies were retained when they established foundational mechanisms or historical benchmarks. The review does not claim systematic, exhaustive, date-stamped, PRISMA-compliant, or meta-analytic coverage.

Titles and abstracts were assessed for relevance, followed by full-text evaluation where available. Backward citation checking was used to identify foundational mechanisms and original studies underlying narrative author citations. Bibliographic information and DOIs of newly added studies were cross-checked against publisher or bibliographic records.

Studies were included when they addressed polysaccharide-based or polysaccharide-composite hydrogels, hydration-mediated antifouling interfaces, resistance to bacterial adhesion, antibiofilm biomaterials, porous hydrogel transport, or application-relevant scaffold and coating performance. Primary studies were prioritized when they reported polymer composition, network or cross-linking strategy, pore structure, swelling, mechanics, wettability or hydration, protein adsorption, bacterial adhesion or biofilm outcomes, cytocompatibility, durability, or in vivo performance. Studies focused exclusively on drug release were excluded unless they provided relevant interfacial, network, infection, or transport evidence; nonbiomedical wetting studies were included only when their methods directly informed dynamic liquid–solid characterization.

The study-level comparison reports only values available in the cited sources for polymer composition, network/cross-linking strategy, pore size or porosity, swelling, mechanical properties, wettability or hydration, bacterial adhesion/antibiofilm performance, test organism, exposure time, and application model. “NR” is used when a requested parameter was not reported; no values were estimated. Because this is a structured narrative synthesis rather than a systematic review or meta-analysis, no PRISMA flow count is claimed.

### 2.2. Four-Layer Interface Framework

The literature is synthesized across four evidence-linked levels. The basic level addresses hydration-mediated resistance to protein and bacterial approaches; the enhanced level addresses cross-linking, mechanical reinforcement, wet-state adhesion, and resistance to flow or abrasion; the regulated level addresses stimulus-responsive or limited active antibacterial functions; and the integrated level addresses host-cell compatibility, inflammation control, vascularization, tissue integration, and long-term service stability. This framework organizes the evidence without replacing study-level comparisons.

## 3. Polymer Network Design of Polysaccharide-Based Composite Hydrogels

From a structure–performance perspective, the anti-infective performance of polysaccharide-based hydrogels depends not simply on the presence of antibacterial components but on whether molecular structure, cross-linking mode, swelling behavior, and interfacial hydration jointly maintain a stable low-fouling interface. Polysaccharides differ in charge, hydrophilic functional groups, cross-linkable sites, degradation behavior, and mechanical support; these differences define their functional boundaries in antifouling, resistance to bacterial adhesion, active regulation, and tissue integration [16,17,18,19,20].

### 3.1. Polysaccharide Molecular Structure and Interfacial Hydration Capacity

Polysaccharides are natural polymers composed of monosaccharide units linked by glycosidic bonds and occur widely in plants, animals, microorganisms, and marine organisms. Their molecular chains often contain hydrophilic functional groups such as hydroxyl, carboxyl, amino, acetamido, or sulfate groups. These groups determine hydration behavior and provide important sites for chemical modification, cross-linking, and composite construction [21].

**Figure 2:**
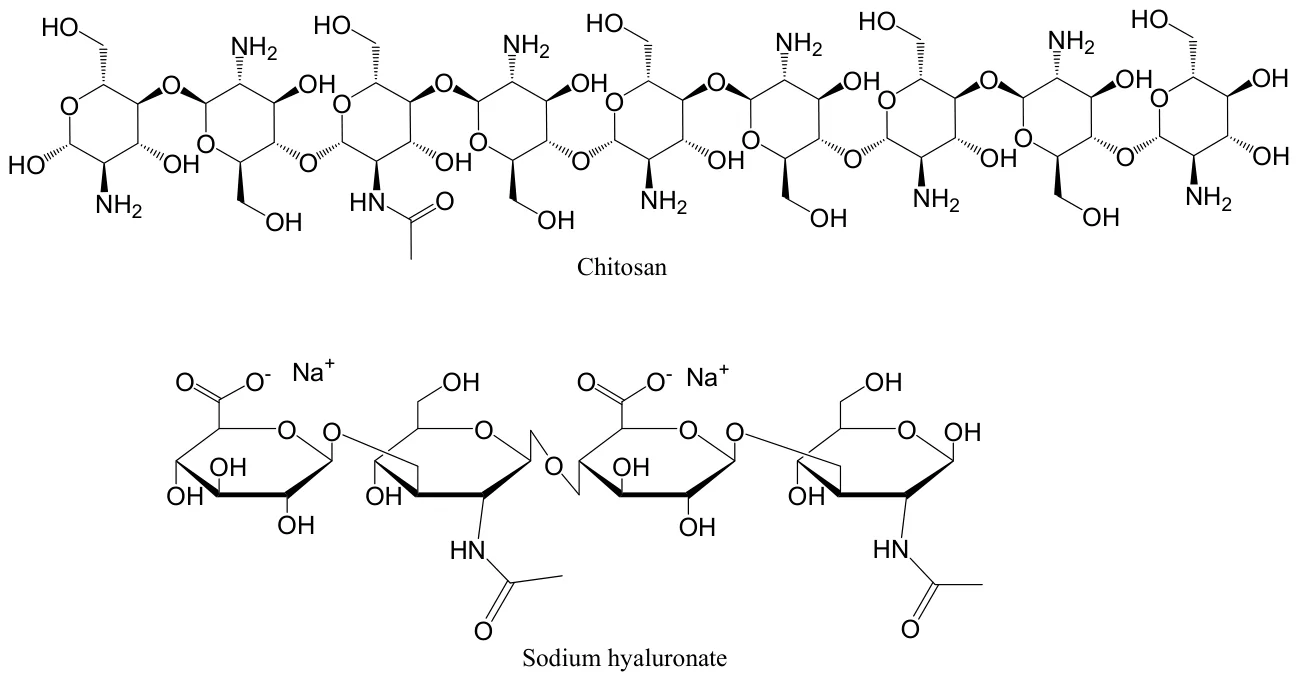
Representative polysaccharide structures and key hydrophilic functional groups of chitosan and sodium hyaluronate.

In antifouling interfaces, hydrophilic functional groups do more than absorb water: through hydrogen bonding, electrostatic, and dipole–dipole interactions, they create interfacial water with different degrees of stability. Water in a hydrogel network can be broadly classified as free, bound, or confined. Free water migrates and is lost readily and contributes little to long-term antifouling, whereas bound and confined water interact more strongly with polymer segments and are more difficult for proteins and bacteria to displace. The antifouling performance of polysaccharide-based hydrogels therefore depends not only on total water content but also on interfacial water stability [22]. Representative molecular structures and key hydrophilic functional groups of chitosan and sodium hyaluronate are shown in Figure 2.

### 3.2. Structural Differences and Functional Boundaries of Typical Polysaccharide Materials

Chitosan is a cationic polysaccharide whose amino groups can be protonated under acidic conditions and interact with negatively charged bacterial surfaces. Its advantages include the high reactivity of its amino groups, facile quaternization, carboxymethylation or graft modification, and the capacity to interact with bacterial membranes. Limitations include pH-dependent solubility; strong cationization may increase protein adsorption, disrupt cell membranes, and raise hemocompatibility concerns [23,24,25]. The key to chitosan-based systems is therefore not simply to increase positive charge density but to establish a controlled design window encompassing antibacterial activity, protein-fouling resistance, and host-cell compatibility. The structural characteristics, principal advantages, limitations, and application boundaries of representative polysaccharides are summarized in Table 1.

Alginate provides mild ionic cross-linking, good fluid absorption, and moisture retention, making it suitable for wound dressings and injectable gels. However, its single ionically cross-linked network may loosen through ion exchange in complex body fluids, leading to uncontrolled swelling and reduced mechanical performance. Hyaluronic acid offers excellent water retention, lubricity, and tissue compatibility and is suitable for soft-tissue repair, lubricating interfaces, and wound materials, but it degrades readily, has limited mechanical strength, and provides insufficient long-term fixation. Cellulose and its derivatives are widely available and structurally stable; nanocellulose can reinforce hydrogels, although its intrinsic antibacterial activity remains limited [40,41]. No polysaccharide is universally advantageous; suitability depends on matching structural characteristics to the intended application. These differences define the application boundaries of polysaccharide materials. In catheter-related applications, studies of hydrophilic and zwitterionic coatings show that protein rejection and resistance to bacterial adhesion must be evaluated together with coating retention under friction and flow. Accordingly, polysaccharide coatings should be assessed not only by initial wettability but also by protein adsorption, bacterial adhesion, and functional retention after dynamic-flow exposure.

### 3.3. Coupling of Cross-Linked Networks, Swelling Behavior, and Mechanical Stability

The interfacial performance of polysaccharide hydrogels is strongly influenced by network cross-linking density. Increasing cross-linking density usually improves mechanical strength, limits excessive swelling, and delays degradation, but it can also reduce the free volume available for hydration, water content, and segmental mobility, thereby weakening interfacial hydration. An excessively low cross-link density promotes water uptake and swelling but may result in a loose network, inadequate mechanical integrity, and interfacial delamination. Interfacial performance does not necessarily improve monotonically with increasing softness, hydrophilicity, or swelling. A suitable design window must balance hydration capacity and structural stability [42].

Pore architecture is governed jointly by cross-linking density, polymer–water interactions, processing history, and the drying or measurement method. Higher cross-linking density generally narrows the effective mesh and suppresses swelling, whereas lower density increases water uptake and molecular transport but can reduce modulus and dimensional stability [43,44]. Pore size, porosity, interconnectivity, and tortuosity determine the effective contact area and the transport of nutrients, oxygen, antibiotics, proteins, and bacterial products. Electron microscopy of dried gels can distort pore dimensions; hydrated-state fluorescence or transport measurements are therefore needed to connect apparent morphology with functional mass transfer [45]. In regenerative scaffolds, interconnected macropores support cell migration and perfusion, but rough recesses and poorly flushed pores can protect bacteria from fluid shear and promote extracellular polymeric substance accumulation. Consequently, pore architecture should be optimized together with swelling, mechanics, hydration retention, and cleanability rather than treated as an independent variable.

## 4. Structure–Performance Relationships: Network Design, Porosity, Mechanics, and Interfacial Function

**Figure 3:**
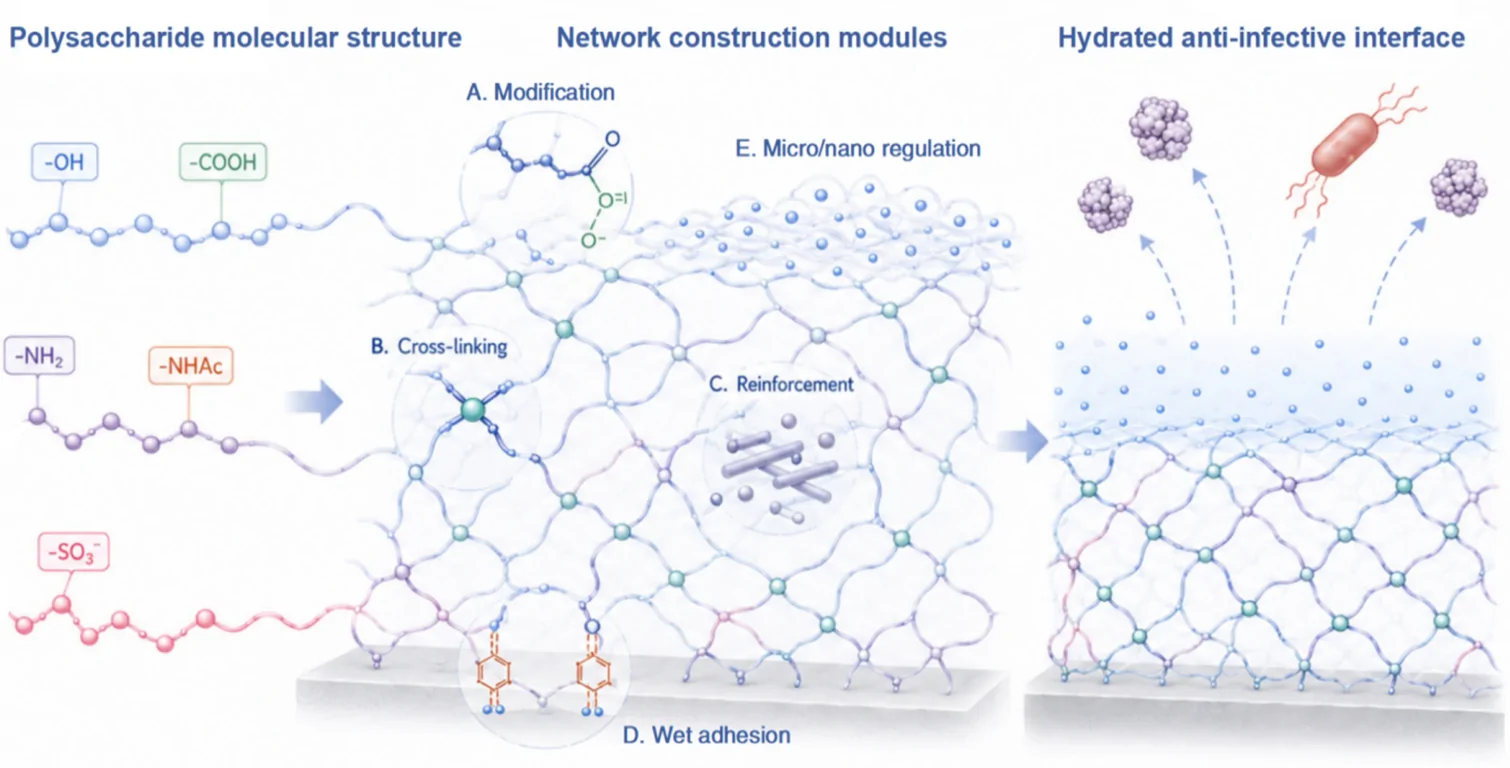
Molecular-to-interfacial design of polysaccharide hydrogel networks for hydrated anti-infective interfaces. Colored polymer chains represent polysaccharides bearing –OH (blue), –COOH (green), –NH_2_ (purple), –NHAc (orange), and –SO_3_^−^ (red) groups. A–E denote modification, cross-linking, reinforcement, wet adhesion, and micro/nano regulation, respectively. Solid horizontal arrows indicate the progression from molecular structure through network construction to formation of the hydrated interface; curved lines depict polymer chains, colored spherical nodes indicate cross-linking or functional sites, and blue dots represent interfacial water. Dashed arrows indicate the repulsion of proteins (purple clusters) and bacteria (red rods) from the hydrated interface.

For polysaccharide-based composite hydrogels, network design should balance competing structure–performance requirements rather than simply accumulate hydrophilic, antibacterial, adhesive, or reinforcing components. Hydrophilic modification favors hydration-layer formation but may weaken mechanical stability and tissue integration when excessive; cationization can enhance bacterial membrane interactions but may increase protein adsorption and cytotoxicity at high charge density; increased cross-linking improves wet-state stability but may reduce swelling and segmental hydration; and micro-/nanostructures can enhance superwetting but may also create local bacterial niches. Network design should therefore establish an application-specific parameter window encompassing hydration-layer stability, mechanical integrity, antifouling and anti-adhesion behavior, and biocompatibility. These coupled molecular-to-interfacial design relationships are summarized in Figure 3.

### 4.1. Molecular Modification and Interfacial Hydration

Polysaccharide modification commonly includes carboxymethylation, quaternization, sulfation, aldehyde functionalization, methacrylation, graft polymerization, and zwitterionization. These modifications are often described as improving solubility, gelation, or antibacterial properties, but their effects on interfacial water structure and the energetic penalty for foulant approach are particularly important for superwetting anti-infective interfaces. For example, carboxymethylation can improve hydration [46], quaternization can enhance bacterial membrane disruption, and zwitterionization can form a more strongly hydrated electrostatic layer. However, excessive charge density or anti-adhesion can alter protein adsorption, increase cytotoxicity, or impair tissue integration [47]. Molecular modification must therefore balance hydrophilicity, charge density, antibacterial performance, and biocompatibility for the intended application.

### 4.2. Cross-Linking Strategies and Dynamic Network Stability

Physical, chemical, and dynamic covalent cross-linking offer distinct advantages. Physical cross-linking uses mild conditions but may provide insufficient long-term stability and erosion resistance. Chemical cross-linking can form a stable covalent network and improve mechanical strength and durability, but residual cross-linkers and reaction conditions require careful biosafety assessment. Dynamic covalent cross-linking can balance stability and reversibility, providing self-healing, injectability, and stress relaxation. For anti-infective interfaces, evaluation should establish both successful gelation and functional retention in dynamic body-fluid environments. Chitosan-based dynamic covalent hydrogels illustrate how injectable, self-healing networks can conform to irregular infected wounds and maintain continuous coverage [48]. Dynamic bonds such as Schiff-base linkages combine gelation with injectability, self-healing, and wet-state adaptability.

### 4.3. Double and Multiple Networks: Addressing the Trade-Off Between High Hydration and High Strength

Double-network and multi-network structures are important strategies for addressing the high-water-content/low-strength trade-off in polysaccharide hydrogels [49]. One network can provide rigid support and energy dissipation, while a second network provides flexibility, toughness, and hydration [50]. This combination can improve tensile, compressive, and failure resistance without abandoning a highly hydrated state. The design window remains constrained: excessive reinforcement can reduce segmental mobility and swelling, while excessive softness can compromise wear resistance and coating retention. The relevant target is therefore simultaneous wet-state mechanical integrity, hydration-layer stability, and interfacial antifouling performance rather than maximum strength alone [51].

### 4.4. Mussel-Inspired Adhesion: Addressing Wet-State Fixation

Insufficient wet-state adhesion is a key obstacle to translating hydrogel coatings from the laboratory to application. Hydrophilic coatings can delaminate in body-fluid environments because of interfacial water, mechanical friction, and flow-induced erosion. Mussel-inspired catechol chemistry can enhance wet adhesion between hydrogels and metals, polymers, ceramics, or biological tissues through hydrogen bonding, metal coordination, π–π interactions, and covalent reactions [52,53,54]. However, catechol chemistry is not a universal solution: oxidation may affect material color, adhesion durability, and interfacial reactivity, while excessive cross-linking may reduce hydrogel flexibility and hydration. Mussel-inspired adhesion should therefore support long-term interfacial stability rather than merely maximize initial adhesion strength [55].

### 4.5. Micro-/Nanostructures: Enhancing Superwetting or Creating Bacterial Niches?

Micro-/nanostructures can amplify the wetting behavior of hydrophilic surfaces and enhance interfacial water stability. Appropriate roughness can improve water capture and support a continuous hydration layer. However, rough structures may also create bacterial niches that reduce exposure to fluid shear and promote local extracellular polymeric substance accumulation [56]. When designing surfaces that resist bacterial adhesion, structural scale must be considered together with bacterial size, morphology, and motility [57]. Effective micro-/nanostructures should stabilize hydration while limiting bacterial, mechanical interlocking and local sheltering.

### 4.6. Nanocomposites: From Functional Stacking to Functional Coupling

Nanocellulose, silica, hydroxyapatite, graphene oxide, metal–organic frameworks, silver nanoparticles, and copper-based nanomaterials can improve the mechanical properties, antibacterial activity, responsiveness, and structural stability of polysaccharide hydrogels. However, nanocomposite design should avoid simple functional stacking [58]. Excessive concentrations of antibacterial nanocomponents may introduce cytotoxicity, inflammatory stimulation, and long-term release risks, while complex composite systems can complicate quality control and regulatory approval. For translatable materials, designs that achieve clear functions with fewer components are generally preferable to multicomponent accumulation.

### 4.7. Balanced Design Window for Structural Parameters

The preceding evidence indicates that no single structural parameter can be maximized independently without compromising other interfacial functions. The beneficial effects, imbalance risks, evaluation indices, and corresponding design principles for key structural parameters are summarized in Table 2.

## 5. Hydration-Layer Stabilization and Antifouling/Anti-Adhesion Mechanisms

### 5.1. Origins of the Hydration Layer: Free, Bound, and Confined Water

The hydration layer is not simply water adsorbed on a material surface; it is an interfacial water structure jointly stabilized by hydrophilic groups, ionic groups, zwitterionic units, and the hydrogel network. Hydroxyl, carboxyl, and amino groups can form hydrogen-bonded hydration layers, while ionized groups can form more strongly hydrated structures through electrostatic interactions [68]. Zwitterionic groups bind water through paired positive and negative charges, creating confined water that is more difficult for proteins to displace. Hydration-layer evaluation should therefore consider not only water content but also interfacial water stability and retention in the presence of salts, serum proteins, and dynamic flow.

At the molecular level, hydration-mediated antifouling does not arise solely from “surface hydrophilicity.” Surface-sensitive spectroscopy of zwitterionic and PEG interfaces links organized interfacial water to low-fouling performance. Accordingly, polysaccharide hydrogel evaluation should extend beyond contact angle and swelling to include the fractions of bound and confined water, hydration retention during competitive protein adsorption, and interfacial stability under dynamic conditions.

### 5.2. How the Hydration Layer Resists Protein Adsorption: Energetic Penalty of Interfacial Water Replacement

Protein adsorption involves approach, interfacial dehydration, conformational rearrangement, and surface binding [69]. On hydrophobic or high-surface-energy materials, weakly associated interfacial water is readily displaced by proteins, which can then adsorb irreversibly [70]. At a stably hydrated interface, an approaching protein must first displace the interfacial water layer, increasing the energetic penalty for adsorption [71]. Stable hydration can also limit protein conformational spreading on hydrophobic surfaces, making the formation of a stable conditioning film less favorable [69].

The protein-fouling resistance of a hydration layer is environment-dependent. Under high ionic strength, competitive serum protein adsorption, and dynamic shear, a weak hydration layer may be compressed, displaced, or disrupted [72]. Hydrophilicity measured only in deionized water or simple buffer does not demonstrate long-term antifouling performance in body fluids. Hydration-layer stability is therefore more informative than initial hydrophilicity [73].

### 5.3. How Hydration Layers Resist Bacteria: Contact Probability, Surface Morphology, and Active Bacterial Disruption

Bacterial adhesion differs from protein adsorption because bacteria are micrometer-scale, motile, or appendage-bearing cells with complex morphology, surface charge, hydrophobic domains, and extracellular polymeric substance production [74]. Resistance to bacterial adhesion therefore depends not only on interfacial water stability but also on whether bacterial structures can penetrate or disrupt the hydration barrier [49]. A stable hydration layer can reduce direct surface contact and weaken hydrophobic and nonspecific interactions; however, local adhesion can still occur if the layer is unstable, or the surface contains sheltering depressions. Hydration-layer thickness alone is therefore insufficient: stability, surface morphology, roughness scale, bacterial size, and fluid shear must be considered together. Evidence from biomimetic superhydrophilic phosphorylcholine coatings further supports the role of stable hydration in reducing direct bacterial contact and initial colonization.

### 5.4. Antibiofilm Formation: Early Blocking Rather than Late Removal

Biofilm development usually includes reversible adhesion, irreversible adhesion, microcolony formation, extracellular polymeric substance accumulation, mature biofilm formation, and bacterial dispersal [50]. Polysaccharide-based superwetting hydrogels act mainly during the first two stages by reducing conditioning-film formation and initial bacterial adhesion [51]. When bacteria cannot attach stably, subsequent microcolony development, matrix accumulation, and biofilm maturation are constrained.

Early interruption offers greater material design value than later disruption of mature biofilms [75]. Mature biofilms contain nutrient gradients, oxygen gradients, extracellular matrix barriers, and dormant cell populations; consequently, simple antimicrobial treatment or agent release often fails to eradicate them completely. Biomimetic superwetting polysaccharide-based composite hydrogels therefore function most appropriately as biofilm-preventive interfaces: their primary role is to reduce the probability of protein-conditioning film formation and initial bacterial colonization rather than to eradicate established biofilms.

### 5.5. Failure Mechanisms of the Hydration Layer

Once formed, a hydration layer is not permanently stable; its failure mechanisms determine whether a biomimetic superwetting polysaccharide hydrogel can progress from short-term antifouling to long-term use. Major failure pathways include displacement of interfacial water by competitive protein adsorption; charge screening by salts [76], which thins the electrostatic hydration layer; disruption by fluid shear and mechanical friction; network loosening, softening, or delamination due to excessive swelling; and penetration of the barrier through active bacterial adhesion and extracellular polymeric substance secretion. Once bacteria establish local colonization and secrete an extracellular matrix, the barrier effect of the hydration layer is substantially weakened.

Effective hydration-mediated antifouling therefore depends less on initial formation than on long-term maintenance of the hydration layer. This requirement explains why anti-infective hydrogels must simultaneously account for polymer network stability, wet-state adhesion, dynamic wear resistance, and competitive protein adsorption.

### 5.6. Correspondence Between Hydration-Layer Mechanisms and Evaluation Metrics

Hydration-layer quality cannot be inferred solely from initial contact angle or swelling. More informative evaluation combines bound/free-water ratios, dynamic contact angle, wettability retention during competitive protein adsorption, surface-energy changes after long-term immersion, and antifouling performance under fluid shear. A low contact angle indicates initial wettability, a high swelling ratio indicates water uptake, and a short-term reduction in protein adsorption does not establish long-term antifouling. For biomimetic superwetting polysaccharide hydrogels, the key questions are whether the hydration layer remains stable in the presence of salts, competing proteins, fluid shear, and mechanical friction, and whether this stability has an interpretable causal relationship with resistance to bacterial adhesion and biofilm formation.

### 5.7. Study-Level Quantitative Comparison

To compare representative systems on a common basis, reported network or cross-linking strategies, pore structure or swelling, mechanical and wetting metrics, and antibacterial or antibiofilm outcomes were extracted without estimating unreported values. The resulting study-level comparison is presented in Table 3, where NR denotes information not reported in the original source.

The comparison shows why a low contact angle or high swelling ratio cannot be used as a universal proxy for anti-infective performance. The BC/PDA/ZIF-8/Ag system combined >3000% swelling with >1 MPa tensile strength and sub-1% bacterial survival, whereas QBC/heparin/gelatin emphasized water management (1476% swelling and >90% retention at 120 h) and an inhibition zone endpoint. BC-nanofiber reinforcement produced a sixfold mechanical increase with >99% antibacterial efficiency, while the MgO_2_@PDA/F127–alginate system provided time-resolved rheology and biofilm evidence but did not report an absolute pore size. These non-equivalent endpoints prevent rank ordering across studies and support standardized reporting of hydrated pore structure, mechanics, serum-conditioned adhesion, flow durability, cytocompatibility, and multi-time-point biofilm outcomes. Direct cross-study comparison remains limited because organisms, inocula, media, sample geometry, exposure times, and endpoint definitions differ among the cited reports.

Representative experimental observations from the literature complement the conceptual mechanisms discussed in this review. Fluorescence and confocal microscopy, live/dead bacterial staining, extracellular polymeric substance staining, SEM imaging, contact angle measurements, protein adsorption assays, and flow-based bacterial adhesion tests provide complementary evidence of whether a hydration layer reduces biological contamination.

## 6. Active Antibacterial Functions, Responsive Regulation, and Repair Integration: Interface Synergy Beyond Passive Antifouling

**Figure 4:**
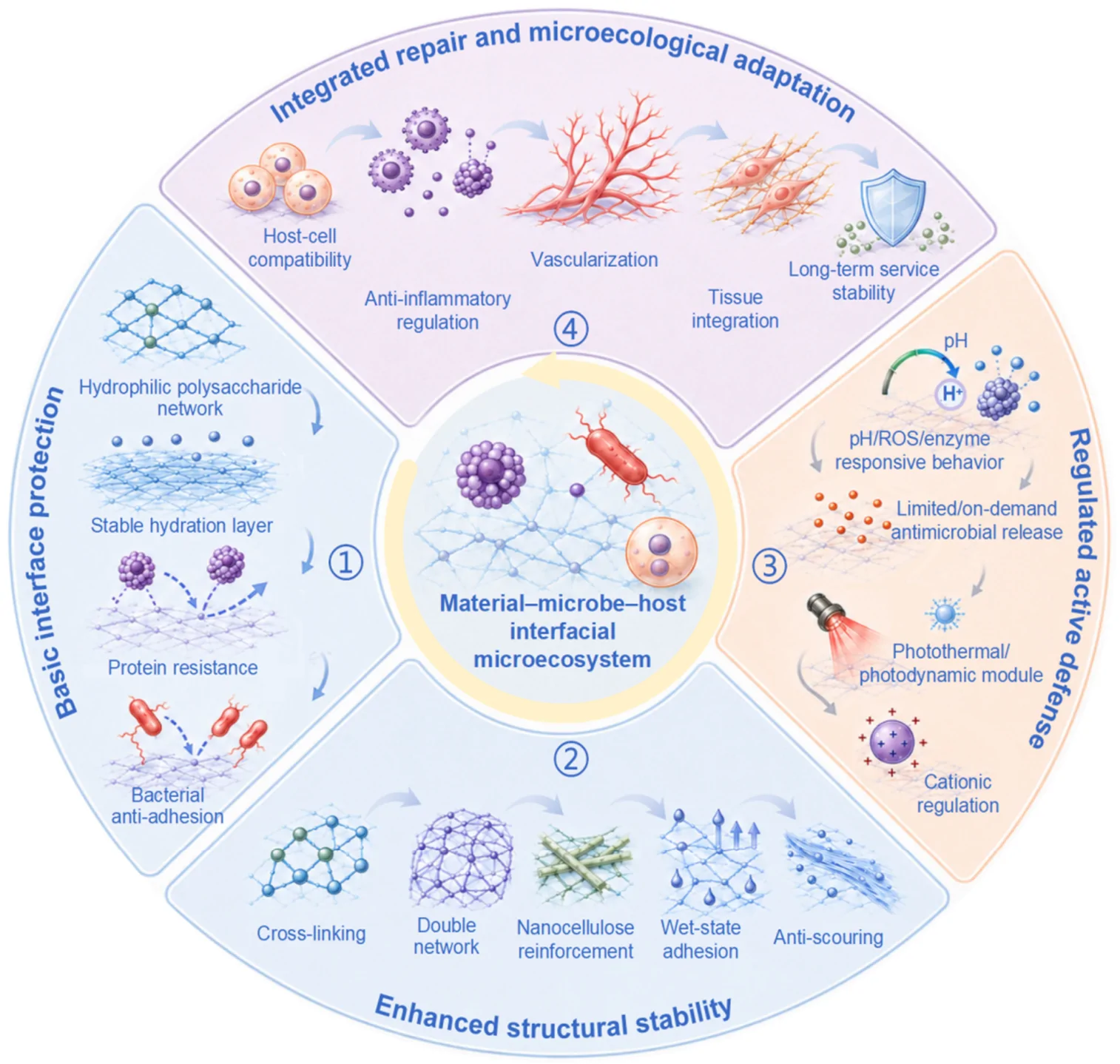
Four-layer design framework of biomimetic superwetting polysaccharide hydrogel anti-infective interfaces. The central circle represents the material–microbe–host interfacial microecosystem. Numbered sectors 1–4 correspond to basic interface protection (blue), enhanced structural stability (light blue), regulated active defense (orange), and integrated repair and microecological adaptation (purple), respectively. The internal arrows indicate increasing functional integration within and among the four levels rather than a mandatory chronological sequence. Network lines represent hydrogel architectures, while the associated icons illustrate representative mechanisms or performance targets within each sector.

Active antibacterial functionality is warranted when it clearly compensates for limitations of passive antifouling [80]. When stable hydration effectively reduces initial adhesion under a low bacterial burden, excessive use of metal ions, photothermal nanocomponents, or strongly cationic structures may increase toxicity and translational complexity. In this review’s framework, active antibacterial activity plays a secondary role and is introduced when passive hydration protection is insufficient, bacterial burden is high, or the infection microenvironment deteriorates [80]. Active components should not disrupt the original hydration layer or low-fouling properties; otherwise, short-term bacterial killing may be achieved at the expense of long-term antifouling. The anti-infective interface can therefore be understood as a four-layer system comprising basic hydration-mediated antifouling, enhanced structural stability, regulated active defense, and integrated repair adaptation, as summarized in Figure 4.

Figure 4 places the material–microbe–host interface at the center and uses clockwise arrows to indicate increasing functional integration rather than a mandatory chronological sequence. Sector 1 (basic interface protection) connects a hydrophilic polysaccharide network to a stable hydration layer, protein-fouling resistance, and resistance to bacterial adhesion. Sector 2 (enhanced structural stability) links cross-linking, double-network construction, nanocellulose reinforcement, wet-state adhesion, and performance under fluid shear to persistence of the hydrated interface. Sector 3 (regulated active defense) contains pH-, reactive oxygen species (ROS)-, and enzyme-responsive release, photothermal or photodynamic modules, and cationic regulation; these functions are intended for limited or on-demand intervention when passive protection is insufficient. Sector 4 (integrated repair and adaptation) links host-cell compatibility, inflammation regulation, vascularization, tissue integration, and long-term service stability. The arrows therefore represent evidence-dependent progression from interfacial hydration to structural durability, regulated defense, and host-compatible repair, while feedback to the central interface emphasizes that failure at any level can compromise the entire system.

Cationic chitosan structures, quaternary ammonium salts, metal ions, and photothermal or photodynamic components can enhance bacterial inactivation, but their dose and spatial range of action must be controlled. If an active antibacterial function causes cytotoxicity, inflammatory stimulation, or accumulation of dead bacteria, it may compromise long-term interfacial stability. For long-term implants or wound repair materials, a high short-term bactericidal rate cannot substitute for sustained biocompatibility and tissue repair quality [81].

A more defensible active-regulation strategy is limited intervention when contamination risk rises rather than continuous release or high-intensity sterilization. Zhang et al. [77] interpenetrated a zwitterionic PSBMA-containing hydrogel network with polymeric substrates and combined passive resistance to bacterial attachment with bacteria-triggered gentamicin release and hyaluronidase-mediated surface renewal. This verified example supports the regulated level of Figure 4, but it also illustrates a translational limitation: responsiveness, drug loading, network durability, and substrate adhesion must all be validated under repeated and long-term exposure.

Responsive interfaces are more selective than continuously active antibacterial systems. Infection-associated changes in pH, ROS levels, enzymatic activity, or temperature can trigger antibacterial, antioxidant, or anti-inflammatory functions [78] while maintaining lower activity during normal repair. The critical design questions are whether the response threshold matches the infection microenvironment, whether the response is reversible, and whether it remains reliable under complex in vivo conditions.

**Figure 5:**
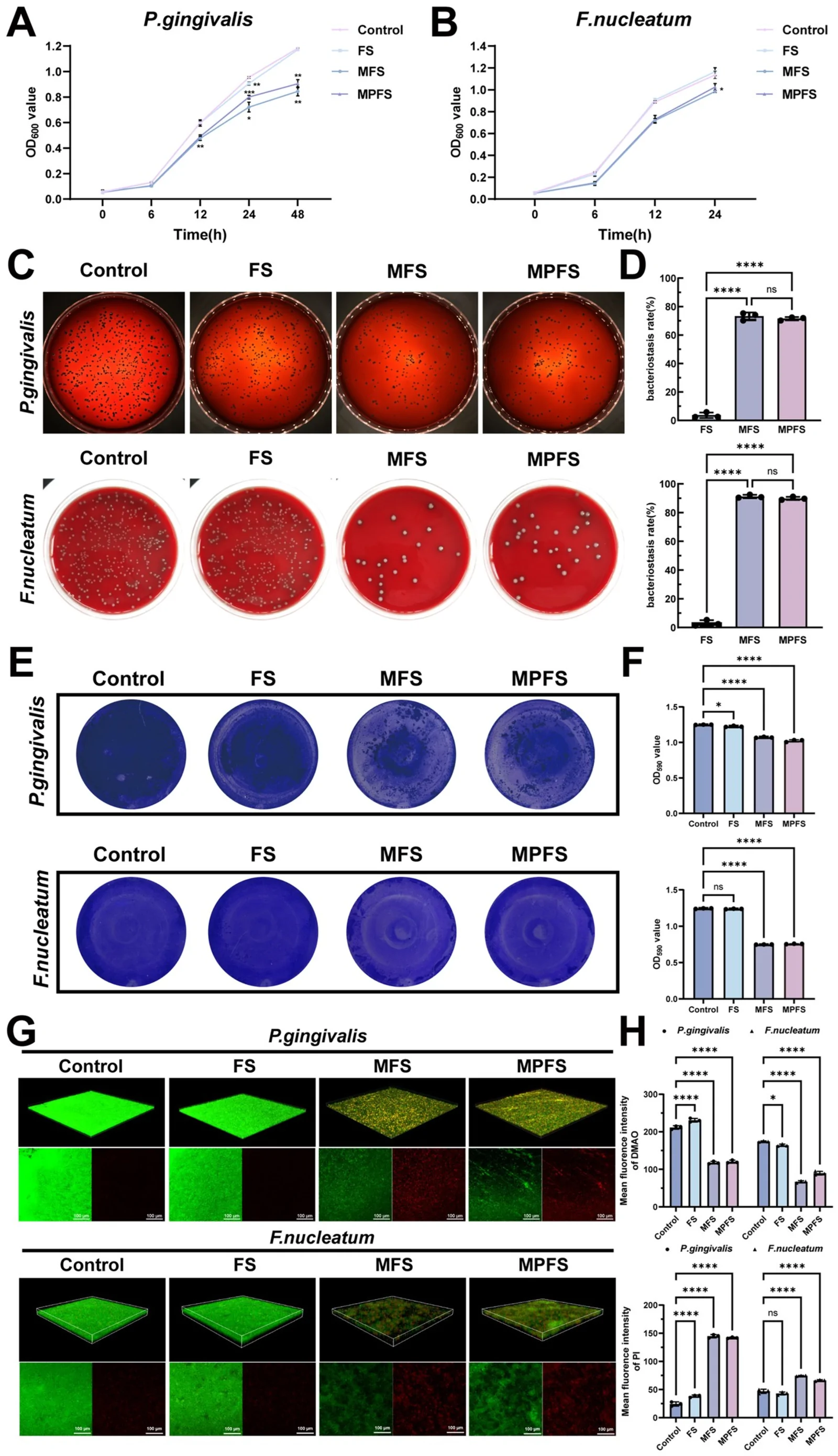
Active antibacterial and antibiofilm performance of MgO_2_@PDA/F127–sodium alginate hydrogel. (**A**,**B**) OD_600_ growth curves for *P. gingivalis* and *F. nucleatum*; (**C**,**D**) colony images and calculated bacteriostasis rates; (**E**,**F**) crystal-violet biofilm staining and OD_590_ quantification; (**G**,**H**) CLSM live/dead biofilm reconstructions and fluorescence quantification. FS denotes the blank F127–sodium alginate matrix, MFS the MgO_2_-containing hydrogel, and MPFS the MgO_2_@PDA-containing hydrogel. Reproduced from Wang et al. [79], Regenerative Biomaterials 2026, 13, rbag098, under CC BY 4.0. All data are presented as mean ± SD (n = 3); ns, not significant; * *p* < 0.05, ** *p* < 0.01, *** *p* < 0.001, and **** *p* < 0.0001.

FS, the blank F127–sodium alginate matrix, showed no significant intrinsic antibacterial activity. The antibacterial and antibiofilm effects observed for MFS and MPFS arose from the MgO_2_/MgO_2_@PDA active component, with the original study assigning the multifunctional response to MgO_2_@PDA rather than to hydration-layer antifouling alone [79]. Figure 5 is therefore evidence for active regulation in Section 6. It also illustrates the value of combining growth kinetics, colony counts, biofilm biomass, and three-dimensional live/dead imaging rather than inferring mechanism from a single endpoint.

Repair-integrating interfaces focus on tissue reconstruction after infection control. Bacterial inhibition alone does not complete tissue repair; failure to reduce excessive inflammation or support reconstruction can still lead to delayed chronic wound healing or implant failure. Future designs should coordinate infection control, inflammation regulation, vascularization, cell migration, and matrix deposition. Active functions should therefore be controllable, low-dose, on-demand, and interfacial while preserving a stable hydration layer and low-fouling interface.

## 7. Application-Specific Design Requirements

Different applications impose distinct requirements on polymer network structure and interfacial performance; antifouling design must therefore be scenario-specific. Catheter coatings and wearable wet interfaces highlight the value of superwetting hydration layers for protein-fouling resistance, low friction, and resistance to bacterial adhesion. Wound dressings must combine antifouling with access for repair-related proteins, cells, and matrix. Implant surfaces and tissue-engineering scaffolds are more complex because the trade-off between resistance to bacterial adhesion and host-cell integration precludes indiscriminate antifouling or complete anti-adhesion.

### 7.1. Wound Dressings: From Drug-Release Dressings to Wet-State Interfacial Regulation

Polysaccharide superwetting wound interfaces should be understood not simply as drug-release dressings but as wet-state interfaces that regulate fouling, protein deposition, bacterial adhesion, exudate management, and the repair microenvironment. Acute wounds emphasize short-term barrier protection and healing speed, whereas chronic wounds often involve persistent inflammation, oxidative stress, poor vascularization, and recurrent bacterial contamination. Wound hydrogels must therefore balance fluid absorption, moisture retention, softness, antibacterial activity, inflammation control, and atraumatic removal [82,83]. The responsive spirulina-protein/carboxymethyl-chitosan emulsion gel reported by Mao et al. [78] combined antibacterial, anti-inflammatory, and pro-vascularization effects in an infected wound environment, illustrating that wound hydrogel design requires coordination among fouling control, inflammatory state regulation, exudate management, and tissue regeneration. Liang et al. [84] developed a dual-functional pin-site dressing that combined a physical, bacterial barrier with a zinc alginate–polyurethane layer for exudate management, achieving bacterial blocking above 95% and approximately 90% inhibition of *S. aureus* and *E. coli*.

### 7.2. Catheters and Long-Term Indwelling Devices: Antifouling, Low Friction, and Secure Coating Attachment

Catheter-related infections usually originate from sustained protein fouling, bacterial adhesion, and biofilm formation on material surfaces [67]. Catheter coatings must tolerate insertion friction, fluid shear, bending, and long-term indwelling. Required properties therefore include resistance to protein and salt deposition, resistance to bacterial adhesion, low friction, secure substrate attachment, and long-term stability. Polysaccharide-based superwetting coatings can reduce fouling and improve lubrication through interfacial hydration, but their practical value is limited if performance cannot be maintained under flow. Wu et al. [85] developed a multifunctional zwitterionic polymer coating on polyurethane ureteral stents using UV-initiated free-radical polymerization combined with dip-coating. The coating resisted 96.1% and 83.5% of encrustation after 30 and 90 days of simulated urine flow, respectively, and reduced bacterial adhesion. Catheter evaluation should therefore extend beyond initial contact angle or short antibacterial assays to hydration retention, coating adhesion, wear, and antifouling performance under flow.

### 7.3. Implant Surfaces: Trade-Off Between Resistance to Bacterial Adhesion and Tissue Integration

The design challenge for implant surfaces is to inhibit early bacterial adhesion while permitting host-cell adhesion, spreading, and tissue integration [86]. A highly antifouling interface may reduce bacterial adhesion but also impede osteoblast, fibroblast, or endothelial-cell attachment, while strongly bactericidal surfaces may cause cytotoxicity or local inflammation. Implant surfaces should therefore move beyond “complete anti-adhesion” or the “highest bactericidal rate” toward time-programmed antifouling layers, degradable antibacterial interfaces, spatially partitioned functions, and cell-selective adhesion.

### 7.4. Tissue-Engineering Scaffolds: Porous Architecture as Both a Regenerative Advantage and an Infection Risk

Tissue-engineering scaffolds must support cell infiltration, nutrient transport, and tissue regeneration and therefore typically have porous architectures with high specific surface areas. These features can also create sites for bacterial retention and biofilm formation [62]. The central trade-off is that structures favorable for cells may also favor bacteria. Excessively large pores, high surface roughness, and abundant protein adsorption sites can promote both cell attachment and bacterial colonization. Scaffold interfaces therefore cannot simply adopt antifouling-coating logic; they require differential responses to mammalian cells and bacteria.

Porous hydrogel architecture deserves special attention because pore size, interconnectivity, tortuosity, surface roughness, and swelling jointly determine regenerative performance and infection risk. Interconnected macropores can promote nutrient diffusion, oxygen transport, waste removal, and cell infiltration, whereas micropores or rough depressions close to bacterial dimensions may protect bacteria from fluid shear and favor extracellular polymeric substance accumulation. For tissue-engineering scaffolds, a useful pore structure should therefore be evaluated not only by SEM morphology or porosity but also by bacterial retention, biofilm thickness, cell infiltration depth, mechanical integrity after swelling, and mass transport under hydrated conditions.

Al-Ahmad et al. [62] compared rapid-prototyped PLGA, PLLA, and PLLA–TCP scaffolds: PLLA and PLLA–TCP supported higher CAL-72 proliferation than PLGA, yet all three scaffolds permitted microbial adhesion; *P. gingivalis* produced the highest CFU counts, and E. faecalis adhered more strongly to PLGA and PLLA than to PLLA–TCP. Yang et al. [63] reported interconnected macropores with nanofibrous walls in 3D bacterial cellulose/agarose scaffolds (dominant pore diameter about 100 μm; porosity 88.5 ± 0.4%), together with C5.18-cell and hBMSC attachment and proliferation. These studies show that pore architectures favorable for cell attachment and growth still require direct assessment of bacterial retention rather than inference from porosity alone.

Compared with collagen/gelatin, polycaprolactone (PCL), poly(lactic acid) (PLA), and PLGA scaffolds, polysaccharide hydrogels offer high water content, abundant modification sites, and mild gelation but generally lower load-bearing capacity and less predictable long-term degradation. Recent bacterial cellulose systems demonstrate the range of achievable properties: BC/PDA/ZIF-8/Ag reached >1 MPa tensile strength and >3000% swelling with strong antibacterial activity [38], BC-nanofiber reinforcement increased self-healing hydrogel strength sixfold [33], and QBC/heparin/gelatin provided 1476% swelling with >90% water retention at 120 h [37]. These gains arise from different active components and test protocols, so they do not establish a universal advantage over PCL, PLA/PLGA, or collagen scaffolds. Polysaccharide networks remain limited by swelling-induced softening, pore structure changes during degradation, batch variability, and the need to preserve cell migration without creating poorly flushed bacterial shelters.

### 7.5. Flexible Medical Interfaces and Wearable Materials: Low-Fouling Stability Under Long-Term Attachment

Flexible medical interfaces and wearable materials contact the skin for extended periods and encounter sweat, sebum, dust, proteins, and microorganisms. Polysaccharide-based hydrogels are attractive because of their softness, water content, and conformability, but water loss, declining adhesion, or excessive skin hydration can reduce long-term comfort [87]. Wearable hydrogels should therefore be evaluated for water retention, reversible adhesion, effects on the skin barrier, and retention of antifouling performance after repeated attachment, rather than for antibacterial performance alone. The differentiated microenvironmental characteristics, performance requirements, failure risks, and design priorities across these application scenarios are summarized in Table 4.

## 8. Characterization of Dynamic Interfacial Processes and Minimum Evidence Standards

Structural characterization should do more than confirm that a material was fabricated; it should test the design hypothesis. Specifically, it should determine whether molecular modification changes functional-group density or charge, whether a stable cross-linked network is formed, whether nanocomponents are distributed uniformly, whether biomimetic adhesive units contribute to interfacial fixation, and whether micro-/nanostructures can be fabricated reproducibly. Without adequate structural evidence, the origins of subsequent antifouling or antibacterial performance cannot be interpreted reliably.

Hydration-layer evaluation should progress from static hydrophilicity to dynamic stability. Contact angle describes initial wettability but not long-term hydration-layer stability. A high swelling ratio does not necessarily indicate strong antifouling performance because it may arise from readily lost free water. More informative evaluation includes the bound water fraction, hydration retention over time, interfacial stability during competitive protein adsorption, and antifouling durability under fluid shear. Based on these considerations, Table 5 summarizes the minimum evidence standards, insufficient evidence patterns, and recommended test conditions for the principal functional claims.

Antifouling evaluation should demonstrate reduced protein adsorption in mixed-protein or serum environments, retention of function after dynamic-flow exposure or long-term immersion, and exclusion of artifacts arising from swelling, surface delamination, or measurement error. A decrease in albumin adsorption alone does not establish long-term antifouling performance in complex body-fluid environments.

Resistance to bacterial adhesion should distinguish among bacteria that are “not adhered,” “dead,” and “washed away.” Anti-adhesion concerns the number of attached bacteria, whereas antibacterial activity concerns survival. If an experiment reports only reduced colony counts without identifying bacterial state and location, the operative mechanism remains uncertain.

Antibiofilm performance cannot be established by a short-term planktonic-bacteria killing assay. Such claims should be supported by reduced early adhesion, lower extracellular polymeric substance abundance or biofilm thickness, and observations at multiple time points that distinguish durable biofilm control from transient bacterial killing. Materials intended for long-term use should also be evaluated for recontamination and biofilm recurrence.

Translational claims should integrate evidence for performance retention after sterilization, wettability and mechanical stability after long-term immersion, antifouling performance after friction and under flow, and cell and blood compatibility. Application-specific models are also required: wound materials should address exudate and tissue repair, catheter materials should address fluid shear and long-term indwelling, implant materials should address competition between bacteria and host cells, and wearable materials should address sweat, sebum and repeated attachment.

Future characterization should move from static endpoint wettability toward methods that answer distinct interfacial questions. LSCM/CLSM and real-time fluorescence imaging resolve where liquid, proteins, bacteria, extracellular polymeric substances, and viable/dead cells are located and how those distributions change with time; Varol and Seeger [96] demonstrated fluorescence-assisted three-dimensional confocal characterization of silicone micro- and nanopatterned surfaces, illustrating how confocal optical imaging can resolve complex surface topography. QCM-D measures time-resolved adsorbed mass together with dissipation, distinguishing rigid protein deposition from hydrated viscoelastic layers. AFM force mapping quantifies local adhesion, modulus, roughness, and interaction forces under liquid. Interfacial rheology measures the time-dependent shear or dilatational mechanics of adsorbed interfacial layers. Sum-frequency generation or related surface-sensitive spectroscopy probes molecular orientation and interfacial water organization. Dynamic advancing/receding contact angles quantify hysteresis, whereas microfluidic flow and real-time bacterial imaging connect attachment, detachment, growth, and biofilm formation to define shear. Fluorescence transport methods can additionally quantify diffusion and confinement in hydrated nanoporous networks [45]. Together, these techniques identify mechanisms that a single static contact angle cannot resolve.

## 9. Clinical Translation Bottlenecks and Future Perspectives

Translational research should report not only peak performance under ideal conditions but also the conditions under which materials fail, whether failure begins in the hydration layer, polymer network, interfacial adhesion or biocompatibility, and whether structural design can delay or repair that failure. For biomimetic superwetting polysaccharide-based composite hydrogels, defining failure modes has greater translational value than maximizing short-term antibacterial rates.

### 9.1. Short-Term Performance Does Not Necessarily Predict Long-Term Effectiveness

Many studies report high short-term antibacterial or anti-adhesion rates under static culture conditions but lack validation in serum-containing media, under fluid shear, after long-term immersion, or after repeated mechanical friction. For materials intended for prolonged contact with body fluids or tissues, clinically relevant endpoints are the duration of functional retention and the mode of failure rather than the peak short-term effect.

### 9.2. Trade-Off Between Hydrophilic Antifouling and Mechanical Stability

High hydrophilicity and water content promote hydration-layer formation but often reduce mechanical strength and durability. Increasing cross-linking density or introducing a reinforcing phase can improve mechanical properties but may limit swelling and hydration-layer formation. A balance should be sought through double networks, gradient structures, dynamic cross-linking, and nanoreinforcement rather than by choosing either hydrophilicity or strength.

### 9.3. Trade-Off Between Anti-Adhesion and Tissue Integration

An antifouling interface can inhibit bacterial adhesion but may also inhibit host-cell adhesion and tissue repair. For implants and tissue-engineering scaffolds, this trade-off is particularly important [33,36,77,79,97]. Future materials should move from nonspecific anti-adhesion toward selective regulation that distinguishes bacterial colonization from host-cell integration across temporal and spatial scales.

### 9.4. Trade-Off Between Multifunctionalization and Manufacturability

Some composite systems integrate multiple polymers, nanomaterials, active components, and responsive units. Although these additions can improve laboratory performance, they also increase compositional complexity, quality control burden, and regulatory uncertainty. Multifunctionalization should favor functional synergy over functional stacking. If each function requires additional components, the material may incur penalties in safety, stability, and manufacturability.

### 9.5. Sterilization, Storage, and Large-Scale Preparation

Hydrogel materials are sensitive to high temperature, irradiation, drying, and chemical sterilization. Sterilization processes may cause network fracture, structural shrinkage, active component failure, or degradation of wetting performance. The favorable performance of freshly prepared laboratory samples may not persist after storage, transportation, and sterilization. Future research should incorporate freeze-drying and rehydration, mild sterilization, packaging stability, and batch-to-batch consistency at the material design stage [95].

### 9.6. Product Attributes and Regulatory Pathways

The regulatory classification of polysaccharide-based composite hydrogels may vary with functional design. Simple antifouling coatings may be regulated as medical devices; systems containing antibacterial drugs or active-release components may follow a drug–device combination pathway; composites containing metal nanoparticles, MOFs, or photothermal components may require additional nanosafety evaluation; and degradable implantable hydrogels require longer-term evidence on degradation products and tissue responses. Translational design should therefore avoid unnecessary functional stacking and reduce regulatory risk through clearly defined composition and mechanisms of action.

### 9.7. Standardized Evaluation and Cross-Study Comparability

Differences in strains, culture conditions, protein models, contact times, washing procedures, and statistical methods limit cross-study comparability. Future evaluation should use more application-relevant systems, including dynamic-flow models, multispecies biofilms, long-term testing under flow, post-sterilization functional testing, and animal infection models. Verification under conditions closer to intended use will improve the reliability and translational value of research conclusions [95].

## 10. Conclusions

The primary value of biomimetic superwetting polysaccharide-based composite hydrogels lies not in simple antimicrobial killing or drug release but in regulating interfacial hydration through polymer network structure and thereby reducing protein adsorption, initial bacterial adhesion, and biofilm formation. Stable hydration distinguishes these materials from traditional contact-killing surfaces and makes them more suitable for low-disturbance infection prevention applications involving long-term contact with tissues or body fluids.

From a materials design perspective, their performance depends on interactions among polysaccharide structure, functional-group density, cross-linking mode, pore size, roughness, modulus, swelling behavior, and composite components. Future research should not maximize a single parameter but should establish a balanced structure–performance–function window that accounts particularly for hydration-layer stability, mechanical integrity, and biocompatibility.

The principal trade-offs in this field are hydrophilic antifouling versus mechanical stability, resistance to bacterial adhesion versus tissue integration, and multifunctionality versus manufacturability. Addressing these trade-offs requires scenario-dependent selective interfacial design rather than indiscriminate antibacterial activity or complete antifouling. Future competitiveness will depend not on increasingly complex multifunctional hydrogels but on balancing stable hydration, reliable antifouling, controllable antibiofilm activity, and translatable manufacturing using the minimum necessary components.

## Figures and Tables

**Table 1:** Structural characteristics and functional boundaries of typical polysaccharide materials in anti-infective interface construction.

Polysaccharide Type	Main Structural Characteristics	Main Advantages	Main Limitations	Application Scenarios and Core Trade-Offs	Ref.
Chitosan	Rich in amino groups; cationizable under acidic conditions	Interacts with bacterial membranes; readily supports quaternization, carboxymethylation, or graft modification	Solubility affected by pH; strong cationization may increase protein adsorption, cytotoxicity, and blood compatibility risks	Wound dressings, antibacterial coatings, responsive hydrogels; need to balance antibacterial activity and biocompatibility	[26,27]
Alginate	Rich in carboxyl groups; forms mildly cross-linked networks with Ca^2+^ ions	Mild gelation, good fluid absorption and moisture retention; suitable for wet environments	Ion exchange may loosen the network, cause uncontrolled swelling, and reduce mechanical performance	Wound dressings, injectable gels, wet barriers; need to balance rapid gelation and long-term stability	[28,29]
Hyaluronic Acid	High water retention, lubricity, and tissue compatibility	Suitable for soft-tissue repair, lubrication interfaces, and wound materials	Degrades readily; limited mechanical strength and insufficient long-term fixation	Wound repair, soft-tissue interfaces, lubricating coatings; need to balance tissue friendliness and structural durability	[30,31]
Cellulose/Nanocellulose	Hydroxyl-rich fibrillar framework; nanocellulose provides high-aspect-ratio reinforcement	Renewable reinforcing phase; supports water retention and network toughness	Limited intrinsic antibacterial activity; aggregation and pore densification require control	Reinforced wound hydrogels and scaffolds; balance reinforcement, swelling, transport, and active component dose	[32,33]
Dextran/ Oxidized Dextran	Rich in hydroxyl groups, can be oxidized to introduce aldehyde groups and form dynamic Schiff base networks	Suitable for constructing self-healing, injectable, and dynamically responsive hydrogels	Limited standalone mechanical properties; composite reinforcement is usually required	Injectable hydrogels, wound dressings; need to balance dynamic reversibility and service stability	[34]
Agarose/ Carrageenan	Thermoresponsive or sulfate-containing structure with favorable gelation	Simple gelation; can form hydrophilic networks and composite matrices	Limited functional sites, weak intrinsic antibacterial ability	Basic hydrogel network, composite carrier; need to balance gelation stability and functional tunability	[35]
Bacterial Cellulose	Interconnected nanofiber network with high water content and modifiable hydroxyl groups	Wet-state scaffold, reinforcement, and high fluid uptake	Native BC is not intrinsically bactericidal; active agents or cationic modification can add toxicity or alter transport	Wound dressings and tissue scaffolds; balance swelling, mechanics, mass transport, and antibacterial loading	[36,37,38,39]

**Table 2:** Structural parameter balance window of biomimetic superwetting polysaccharide-based composite hydrogel anti-infective interfaces.

Structural Parameter	Beneficial Effect	Excessive or Imbalance Risk	Recommended Evaluation Index	Corresponding Design Principle	Ref.
Hydrophilic Functional-Group Density	Enhances hydrogen-bonded hydration, improves interfacial hydrophilicity, and reduces the probability of protein approach	Excessive density may alter degradation, mechanical properties, and cell adhesion	FTIR, XPS, contact angle, and bound water fraction	Prioritize stable hydration rather than initial hydrophilicity	[59]
Charge State/Charge Density	Cationic charge enhances bacterial membrane interactions; anionic and zwitterionic charge enhances hydration and antifouling	Excessive cationic charge may increase protein adsorption and cytotoxicity; excessive anti-adhesion may impair tissue integration	Zeta potential, XPS, ionic strength response, and protein adsorption	Establish a charge window that balances antibacterial activity, antifouling, and compatibility	[60]
Cross-Linking Density	Improves mechanical strength, limits swelling and flow-induced erosion, and supports service stability	Excessive density restricts segmental mobility and hydration-layer formation; insufficient density causes network loosening and delamination	Swelling ratio, gel fraction, rheology, and compression/tensile modulus	Balance hydration capacity and structural stability	[61]
Pore Size/Pore Structure	Supports fluid absorption, mass transfer, tissue adaptation, and cell migration	Oversized pores may become bacterial niches and biofilm formation sites	SEM, pore size distribution, fluid absorption rate, and bacterial retention testing	Match pore architecture to bacterial dimensions and the intended application	[62,63]
Surface Roughness/Micro-/Nanostructure	Amplifies hydrophilicity, enhances water capture, and promotes superwetting	Rough depressions may promote bacterial, mechanical interlocking and extracellular polymeric substance accumulation	AFM, SEM, contact angle hysteresis, and bacterial adhesion quantification	Avoid bacterial niches while stabilizing the hydration layer	[64]
Modulus/Mechanical Properties	Maintain coating integrity, improve friction resistance and anti-falling off	An excessively high modulus may cause tissue mismatch. An excessively low modulus promotes deformation and structural instability	Compression/tensile test, rheology, fatigue test	Match mechanical properties to wounds, catheters, implants, and other intended uses	[65]
Wet-state Adhesion Strength	Improves coating fixation, resistance to fluid shear, and long-term service stability	Excessive adhesion may cause injury during dressing changes or intensify interfacial reactions	Lap-shear and peel strength, wet-state friction, and fatigue testing	Optimize adhesion for long-term stability	[66]
Hydration-Layer Stability	Inhibits protein adsorption and initial bacterial adhesion; provides the basis for antifouling	Weak hydration layers are readily disrupted by proteins, salts, fluid shear, and bacterial extracellular polymeric substances	Bound/free-water ratio and dynamic contact angle	Shift from initial hydrophilicity to long-term hydration maintenance	[67]

**Table 3:** Study-level comparison of representative polysaccharide-containing hydrogel systems (NR, not reported in the source).

Study/System	Network or Cross-Linking Strategy	Pore Structure and Swelling	Mechanical/Wetting Metric	Antibacterial or Antibiofilm Test/Result	Ref.
Zhang et al. [77], 2022; HAMA/OHA/PSBMA–gentamicin coating	Interpenetration with polymer substrate; enzyme/pH-responsive self-renewal	NR	PBS durability reported to 30 d; quantitative modulus/wetting NR	*E. coli* and *S. aureus*; 24 h assays; quantitative reduction NR in accessible report	[77]
Mao et al. [78], 2025; spirulina protein isolate/carboxymethyl chitosan emulsion gel	pH/enzyme-responsive curcumin release network	NR	NR	Infected-wound model; quantitative antibacterial value NR	[78]
Wang et al. [79], 2026; MgO_2_@PDA/F127–alginate	Thermosensitive F127–SA matrix containing 1% (*w*/*v*) MgO_2_@PDA	MPFS denser and less porous than FS; absolute pore size NR	Sol–gel transition 35.6 °C; gelation within 135 s at 37 °C	*P. gingivalis* and *F. nucleatum*, 24–48 h; bacteriostasis about 70–90%; live bacteria fluorescence reduced about 50% and 60%	[79]
Deng et al. [36], 2024; dialdehyde BC/quaternized chitosan	Covalent DBC/QCS network with repeatable rehydration	Repeated swelling reported; ratio NR	NR	Antibacterial activity reported; organism-specific value NR	[36]
Li et al. [33], 2024; Ag–BC nanofiber/Res/CND hydrogel	BC-nanofiber reinforcement in a self-healing multifunctional network	NR	Mechanical strength increased sixfold	*S. aureus* 99.99% and *E. coli* 99.68% antibacterial efficiency; infected wound healing shortened from 21 to 14 d	[33]
Liu et al. [37], 2024; QBC/heparin/gelatin	EDC/NHS cross-linking of quaternized BC with heparin/gelatin	3D porous mesh; swelling 1476%; water retention >90% at 120 h	Water vapor transmission 3296 g m^−2^ 24 h^−1^; modulus NR	*S. aureus* inhibition zone 3 cm; test duration NR	[37]
Deng et al. [38], 2023; BC/PDA/ZIF-8/Ag	BC network loaded with PDA/ZIF-8/Ag	Swelling > 3000%	Tensile strength >1 MPa; 50 °C reached in 5 min under NIR	*E. coli* and *S. aureus* survival 0.85% and 0.39%, respectively	[38]
Guamba et al. [32], 2023; cellulose wound hydrogel	Cellulose-derived hydrogel network	NR	NR	Gram-negative bacteria; in vitro/ex vivo antimicrobial effect reported; numeric value NR	[32]
Zhang et al. [39], 2022; GOx/MOF–BC hydrogel	BC-reinforced self-healing gel carrying a glucose-responsive catalytic nanoreactor	NR	NR	Glucose-triggered catalytic antibacterial and hemostatic activity; numeric value NR	[39]

**Table 4:** Differentiated design requirements of polysaccharide superwetting anti-infective interfaces in different application scenarios.

Application Scenario	Main Microenvironment Characteristics	Key Performance Requirements	Main Failure Risks	Design Focus	Ref.
Wound Dressings	Coexistence of exudate, inflammation, bacterial contamination, and tissue repair	Fluid absorption, moisture retention, softness, antibacterial activity, low irritation, and atraumatic removal	Excessive swelling, injury during dressing removal, and inhibition of repair-related proteins or cells	Limit harmful biological contamination while permitting repair-related biological interactions	[84]
Catheters/Long-Term Indwelling Devices	Fluid shear, protein fouling, salt deposition, and ascending-infection risk	Protein-fouling resistance, low friction, resistance to fluid shear, firm coating, long-term stability	Coating delamination, friction injury, and attenuation of antifouling performance	Wet-state adhesion, covalent fixation, wear-resistant network, and dynamic fluid evaluation	[88]
Implant Surfaces	Competitive adhesion between bacteria and host cells, accompanied by immune response and tissue integration	Resist early bacterial colonization while allowing host-cell adhesion and tissue integration	Excessive antifouling inhibits cell adhesion; strong bactericidal activity causes toxicity or inflammation	Selective regulation, temporally programmed responses, and spatially partitioned functionalization	[89]
Tissue-Engineering Scaffolds	Porous architecture and high specific surface area are required for 3D cell growth and mass transfer	Support cell entry, mass transfer, and tissue regeneration; reduce bacterial colonization	Pores become bacterial niches and biofilm formation sites	Control pore size and roughness while promoting differential responses of host cells and bacteria	[63]
Wearable/Flexible Interfaces	Sweat, sebum, skin flora, repeated attachment, and long-term wet contact	Soft, breathable, low irritation, antifouling, reversible adhesion, stable after repeated use	Water loss, declining adhesion, and skin barrier damage	Moisture retention, reversible adhesion, low irritation, and antifouling retention after repeated attachment	[90]

**Table 5:** Minimum evidence standards for biomimetic superwetting polysaccharide-based composite hydrogel anti-infective interfaces.

Claimed Function	Minimum Evidence Required	Insufficient Evidence to Prove Alone	Recommended Test Conditions	Ref.
Successful Material Structure Construction	Functional-group modification, cross-linked networks, nanocomponent distribution, micro-/nanostructures, and adhesion units are present as designed	Gel appearance, a single FTIR peak, or a single SEM image	FTIR, XPS, NMR, SEM/AFM, rheology, element mapping	[91]
Stable Hydration Layer	Increased bound water fraction and retained hydration after exposure to serum, salts, or fluid shear	Low contact angle, high swelling ratio, or short-term wetting image	Dynamic contact angle, DSC/TGA, QCM-D, and wettability retention after serum exposure	[92]
Protein-fouling Resistance	Reduced protein adsorption in mixed-protein or serum environments and retention after dynamic-flow exposure	Single reduction in BSA adsorption	BSA, fibrinogen, fibronectin, or serum protein adsorption under dynamic flow	[92]
Resistance to Bacterial Adhesion	Reduced surface-adherent bacteria with distinction among nonadherent, dead, and detached cells	24 h colony decrease, single viable count	CLSM, SEM, live/dead staining, and bacterial adhesion testing after exposure to fluid shear	[49,73]
Antibiofilm Formation	Reduced early adhesion, extracellular polymeric substance abundance, or biofilm thickness across multiple time points	Planktonic bacteria inhibition, inhibition zone, or short-term bactericidal rate	Crystal violet, CLSM, biofilm thickness, EPS staining, multi-strain model	[93]
Long-term Service Stability	Maintained wettability, mechanical and antifouling performance after immersion, shear, friction, bending	One-time test of fresh samples	Long-term immersion, dynamic-flow testing, friction and wear, and fatigue testing	[91]
Biological Safety	Acceptable cell compatibility, blood compatibility, inflammatory response	Single cell-viability measurement	Multi-cell model, hemolysis, coagulation, inflammatory factors	[94]
Translational Feasibility	Performance retention after sterilization, storage, and scale-up	Performance of freshly prepared laboratory samples	Performance after sterilization and freeze-drying/rehydration; batch consistency; coating adhesion strength	[95]

## Data Availability

No new data were created or analyzed in this review. Data sharing is not applicable to this article.
